# Investigating the electrochemical properties of poly(vinylidene fluoride)/polyaniline blends doped with lithium-based salt

**DOI:** 10.1016/j.heliyon.2024.e37757

**Published:** 2024-09-12

**Authors:** Mahdi Kargar-Esfandabadi, Marzieh Golshan, Hossein Roghani-Mamaqani, Mehdi Salami-Kalajahi

**Affiliations:** a Faculty of Polymer Engineering, Sahand University of Technology, P.O. Box 51335-1996, Tabriz, Iran; b Institute of Polymeric Materials, Sahand University of Technology, P.O. Box 51335-1996, Tabriz, Iran

**Keywords:** Electrochemical properties, Lithium-ion conductivity, Polyaniline, Poly(vinylidene fluoride)

## Abstract

Conductive polymers have attracted much attention in various applications, owing to their excellent chemical, thermal, and oxidative stability. However, they have low dielectric constant, which limits their performance in electrochemical devices. To overcome this drawback, blending with other polymers helps improving their electrochemical properties. Herein, we investigate structural and electrochemical properties of poly (vinylidene fluoride) (PVDF)/polyaniline (PANI) blends doped with lithium-based salt. Results showed that the blends exhibit phase separation of PANI and PVDF, which is confirmed by the thermodynamic interaction parameter. We found that the interaction between the two polymers enhanced the ionic conductivity from 4.9 × 10^−5^ S cm^−1^ for neat PVDF to 5.3 × 10^−4^ S cm^−1^ for composition of 50:50 (PANI50), whereas the ionic conductivity was inversely proportional to the temperature. Moreover, by adding lithium salt to the blend, the thermal stability increased from 376.6 to 478.5 °C for PANI50. The ionic conductivity of the blends depends on the PVDF content, which affects the interaction between the two polymers.

## 1 Introduction

Conductive polymers (CPs) have been utilized in various applications due to tunable morphological features, fast doping and de-doping ability, and charge-discharge kinetics [1], corrosion inhibition due to suitable adhesion to metal surfaces [2], photocatalytic applications due to electrical properties, high theoretical capacitance, high redox activity [3], excellent electrochemical behavior [4], and biomedical and antimicrobial applications [5]. Polyaniline (PANI) is a conductive polymer that has advantages over other conductive polymers in terms of ease of synthesis, low cost of monomer, tunable properties, and better stability [6]. It has various applications such as serving as an emissive layer or a hole-transporting layer in organic light emitting diodes (OLEDs) owing to its luminescence and charge transport properties [7], acting as a sensing material for detecting gases [8], pH [9], humidity [10,87], temperature [11], glucose [12], functioning as a catalyst support [13] or a membrane material [14] in fuel cells because of its proton conductivity, and electrochemical activity, being used as a conductive ink or a thin film for fabricating electronic devices such as printed circuit boards [15], field-effect transistors [16], and memory devices [17] due to its low cost, flexibility, and compatibility with various substrates [18], being employed as a packaging material because of its antimicrobial activity, biocompatibility, and color change properties [19].

Poly (vinylidene fluoride) (PVDF) belongs to the class of insulators [20] and has a wide range of applications in various domains. Its piezoelectric properties allow it to function as a living tissue scaffold that supports electrical stimulation of cells [21]. Its piezoelectric and pyroelectric properties make it suitable for energy producing devices that transform mechanical or thermal energy into electrical energy [22]. Its capability to convert a mechanical variable quantity into a measurable electrical quantity qualifies it for sensors and actuators [23]. Its toughness and resistance to sunlight recommend it for wire coating that offers durable and long-lasting finishes for exterior metal siding [24].

The drawbacks of PANI are its low mechanical strength and thermal stability, which limits its applications in various fields. To overcome this issues, PANI can be blended with other polymers [25]. The PVDF/PANI blend compared to PVDF demonstrates higher β-phase PVDF [28]. At the interface of the PVDF/PANI blend, the charge carriers are depleted due to the difference in conductivity of the polymer phases [26]. Moreover, the dielectric constant of the blend decreases at higher frequencies, because the dipoles cannot align with the electric field. By increasing the amount of PANI in the blend membrane, the ion exchange capacity and the fixed ion concentration are enhanced [27,28]. This is due to the presence of more functional groups, such as amine groups [29,30]. The dielectric constant of PVDF/PANI blend film increases with the PANI content. The dielectric constant at low frequency does not establish interfacial polarization effect that shows the two polymers do not mix well and have different charge carrier relaxation times. This means the charges cannot move easily across the interface of the two polymers [31].

Although the different properties of PVDF/PANI blends have been investigated in literature, these studies have discussed dielectric, mechanical, and thermal properties. Also, in different studies, the content of PANI was very low. In this regard, there is no remarkable work on electrochemical properties of PVDF/PANI blends even in high PANI content. Herein, we have prepared PVDF/PANI blends with different compositions and investigated their properties including crystalline structure, thermophysical properties, thermal stability, and morphological features. We have also utilized different electrochemical analyses to investigate the impedance behavior, electrochemical oxidation-reduction reactions, electrochemical stability window, etc.

## 2 Experimental methods

### 2.1 Materials

Aniline (Merck, 99 %), ammonium persulfate (APS, Merck, 99 %), methanol (Merck, 99.9 %), acetone (Merck, 99 %), lithium hexafluorophosphate (LiPF_6_, Sigma-Aldrich, 99.9 %), diethyl carbonate (DEC, Sigma-Aldrich, 99 %), dimethyl carbonate (DMC, Sigma-Aldrich, 99 %), lithium cobalt (III) oxide (LiCoO_2,_ Sigma-Aldrich, 99.8 %), poly (vinylidene fluoride) (PVDF, kynar761, Arkema), *N*-methyl pyrrolidone (NMP, Sigma-Aldrich, 99.5 %) and carbon black (C, Sigma-Aldrich, 99.9 %) were used as received.

### 2.2 Synthesis of PANI

Aniline (1.0 g, 11 mmol) was dissolved in distilled water (60 mL). Afterwards, 20 mL APS solution (1 M, 10 mmol) was incorporated to the reaction medium, and the reaction was sustained at 25 °C for 5 h. The ensuing mixture was filtrated and rinsed thoroughly with methanol and distilled water. Ultimately, the polyaniline product was desiccated in a vacuum oven at 60 °C for 24 h. The reaction yield was ∼60 % by gravimetric assessment [32].

### 2.3 Preparation of PVDF/PANI bled films

0.2 g PANI was added to 2 mL N-methyl-2-pyrrolidone (NMP) as solvent and 1.8 g PVDF dissolved in NMP for 10 min. Then, two mixture were mixed and stirred to obtain a homogenous mixture. Finally, the mixture was cast into a Teflon mold and dried in an oven at 60 °C. In the same way, other compositions of PANI/PVDF blends (20/80 to 50/50) were prepared. The samples were coded as PANIx where x denotes for the *wt*. % of PANI in the blend.

### 2.4 Preparation of lithium salt-included PVDF/PANI bled films

To prepare blends doped by lithium salt, the films composed of various ratios of PANI and PVDF were immersed in 1 M solution of LiPF_6_ salt in a mixture of DMC and DEC with a volume ratio of 1:1. The films were allowed to swell for 1 h in the electrolyte solution before performing electrochemical characterizations [33].

### 2.5 Characterizations

The ATR spectra of the samples were obtained on a Bruker Tensor 27 F T-IR spectrophotometer in the wavenumber region of 500–4000 cm^−1^ with a resolution of 4 cm^−1^. The samples place on the Zinc selenide (ZnSe) crystal for the analysis. The XRD patterns of the samples were recorded on a Siemens D5000 X-ray diffractometer with a Cu target (λ = 154.0 μm) at room temperature. The instrument operated at 35 kV and 20 mA with a rotating anode generator. The samples were scanned from 2θ = 10–70° in the step scan mode. The DSC curves of the prepared films were measured on a DSC instrument (NETZSCH DSC 200 F^3^, Netzsch Co., Bavaria, Germany) under a nitrogen atmosphere at a flow rate of 50 mL min^−1^. The sample (10 mg) was sealed between aluminum pans and heated from −80 to 200 °C at a heating rate of 10 °C min^−1^ after erasing the thermal history. The TGA curves of the samples were obtained on a thermal gravimetric analyzer (Polymer Laboratories, TGA 1000, UK) under a nitrogen atmosphere at a flow rate of 50 mL min^−1^. Samples (about 10 mg) were heated from room temperature to 800 °C at a heating rate of 10 °C min^−1^. The SEM images were taken by a TESCAN MIRA 5 FE-SEM instrument. The samples were freeze-dried and coated with gold before the characterization.

### 2.6 Electrochemical characterizations

The cathode (LiCoO_2_) and the anode (graphite) were prepared for electrochemical tests as described below. The cathode was composed of 80 *wt* % LiCoO_2_, 5 *wt* % PVDF, and 15 *wt* % super-P carbon black. These materials were dissolved in NMP, and 1.2 mg/cm^3^ of cathodic component was coated on aluminum foil and vacuum-dried at 80 °C for 24 h. The anode was composed of 80 *wt* % graphite, 5 *wt* % PVDF, and 15 *wt* % carbon black. These materials were dissolved in NMP \, and the resulting slurry was coated on copper foil and vacuum-dried at 80 °C for 24 h.

Electrochemical impedance spectroscopy (EIS), linear sweep voltammetry (LSV), and cyclic voltammetry (CV) of the samples were performed using a Potentiostat Galvanostat-PGE 18 (IRASOL) at electrochemical workstation. The EIS measurements were conducted in a frequency range from 0.1 Hz to 1 MHz with an AC potential amplitude of 10 mV. The samples were sandwiched between two stainless steel (SS) sheets (dimension: thickness 0.1 cm, cross-sectional area 0.36 cm^2^) to form block-type cells (SS/sample/SS). The temperature dependence of ionic conductivity was also investigated by performing EIS at 25, 35, 45, and 55 °C. The CV curves of the LiCoO_2_/GPE/graphite cell were recorded by sweeping the potential from 0.0 to 5.0 V at a scan rate of 0.1 mV s^−1^. The transference number (t^+^) of the samples was measured by DC polarization and impedance analysis on the graphite/sample/graphite cell using chronoamperometry with a 5 mV DC voltage.

## 3 Results and discussion

**Fig. 1:**
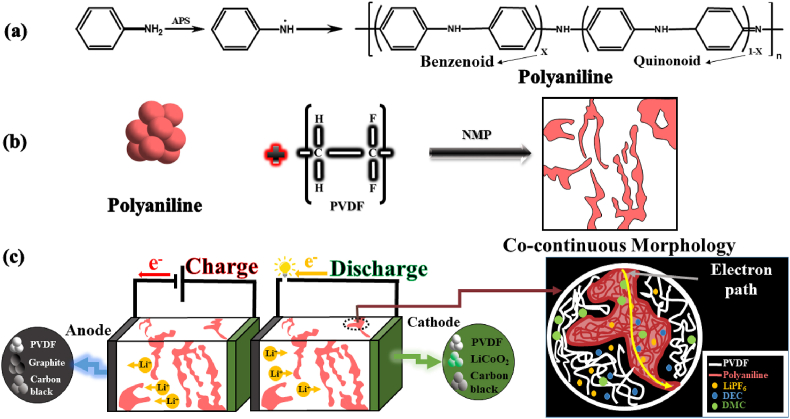
(a) Molecular structure of PANI, (b) Preparation of blend films, and (c) fabrication of electrochemical cell.

PANI is a conductive polymer that can be used as an electrode material in rechargeable battery applications [34]. To synthesize PANI, we used ammonium persulfate (APS) as initiator and oxidant in a chemical polymerization reaction. Next, we prepared blend films of PANI/PVDF, as polymer electrolytes, by casting approach. The blend films were then soaked in a liquid electrolyte solution containing 1 M LiPF_6_ dissolved in a mixture of DEC and DMC with a 1:1 vol ratio. The films were dried and cut into circular pieces, which were sandwiched between a graphite-based anode and a LiCoO_2_-based cathode to form coin cells. We evaluated the electrochemical performance of the cells by measuring their impedance, electrochemical stability window, and ability to dissociate and transfer Li^+^ ions. The schematic diagram of the fabrication process of the PVDF/PANI blends doped by lithium salt is shown in Fig. 1.

### 3.1 Characterizations

**Fig. 2:**
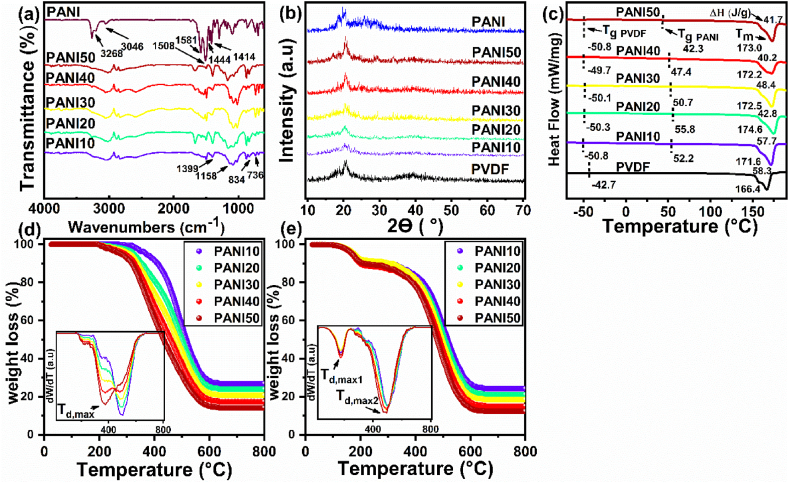
(a) ATR spectra, (b) XRD patterns, (c) DSC curves, and TGA curves (d) before swelling and (e) after swelling for PVDF/PANI blend films.

ATR, XRD, TGA, and DSC were employed to evaluate the structural and thermophysical properties of prepared blends as results are shown Fig. 2a-d. ATR spectra of the samples revealed the characteristic vibrational modes of polyaniline, which are associated with the different types of bonds and ring structures in the polymer backbone. As shown in Fig. 2a, the peaks at 1414 cm^−1^, 1444 cm^−1^, 1508 cm^−1^, 1581 cm^−1^, 3046 cm^−1^, and 3263 cm^−1^ corresponded to the stretching vibrations of C-N, C=N [35], C=C quinocide, C=C benzenoid, C-H, and N-H bonds [36], respectively. The symmetrical stretching vibrations of CF_2_ and CH_2_ groups in PVDF appeared at 1158 cm^−1^ and 1399 cm^−1^, respectively [37]. Fig. 2b shows the XRD patterns of neat PANI, PVDF, and blends with different ratios of PANI. PANI exhibited peaks at 2θ = 14.9, 19.6, and 25.9°, corresponding to the (020), (021), and (200) lattice planes, respectively [38]. The crystallite size was calculated by Scherrer equation as follow:

(1) $$D=\frac{K\lambda }{B\;\cos\;\theta }$$

where *K*, λ, *B* and *θ* demonstrate Scherrer constant, wavelength of the X-ray, the peak width at half of the maximum intensity, and diffraction angle, respectively [39]. The percentage of crystallinity was determined by dividing the area under the crystalline peaks by the area under all peaks that contain the plural of crystalline peaks of diffraction $A_{c}$ and the area of amorphous peaks of diffraction $A_{a}$ from Equation (2) [40].

(2) $$\chi_{c}=\frac{A_{c}}{A_{c}+A_{a}}$$

From the XRD pattern, the degree crystallinity and crystallite size of polyaniline were determined 14.4 % and 44.5 nm, respectively. In PVDF, the peaks at 2θ = 17.9, 18.5, and 20.1° belong to the α phase, and the peak at 2θ = 20.1° indicates the β phase [41]. Due to the overlap of the peaks of the two polymer components, it is not possible to determine the percentage of α and β phases and degree of crystallinity in the blend films. The DSC curves in Fig. 2c show that the pure PVDF has a glass transition temperature (T_g_) of −42 °C. However, in the blends, there are two T_g_ values, one corresponding to PANI and the other to PVDF, which are observed at around 50 and -50 °C, respectively. This confirms that the blend is immiscible [42]. To understand the thermodynamics of immiscibility, compatibility was measured by the interaction parameter. By using Equation (3), the value of the interaction parameter can be determined [43]:

(3) $$\frac{1}{T_{m}}=-\frac{RV_{2u}}{\Delta H_{fu}V_{1u}}\chi_{12}\upsilon_{1}^{2}+\frac{1}{T_{m}^{0}}$$

where $V_{1u}$ is the molar volume of the repeating units in PANI (91.30 cm^3^ mol^−1^), R is the universal gas constant (1.99 cal mol^−1^ K^−1^), $\Delta H_{fu}$ is the molar heat fusion of the repeating units in PVDF (1.60 kcal mol^−1^), $T_{m}$ is the melting point (K) of PVDF crystal in the mixture obtained by DSC (Fig. 2c), $T_{m}^{0}$ is the melting point of pure PVDF equal to 439.5 K (measured by DSC), $\chi_{12}$ is the interaction parameter, and $\upsilon_{1}$ is the volume fraction of PANI. The value of the interaction parameter in the polymer blends is presented in Table 1. In addition, the critical value of the interaction parameter is calculated using Equation (4). If the obtained values of the interaction parameters are greater than this value (*χ*_cr_), the blend becomes immiscible [44].

(4) $$\chi_{cr}=\frac{1}{2}{\left(\frac{1}{\sqrt{N_{1}}}+\frac{1}{\sqrt{N_{2}}}\right)}^{2}$$

*N*_1_ and *N*_2_ represent the degree of polymerization of polymers 1 (PANI) and 2 (PVDF), respectively which are considered 1000 for PANI [45] and 1094 for PVDF, respectively. Therefore, the critical value of the interaction parameter is 0.0019. Since the calculated values of the interaction parameters are greater than this value, it indicates that the prepared blends are immiscible. Additionally, with an increase in the weight percentage of PANI, the value of the interaction parameter decreases from 15.1 to 0.5, indicating a higher miscibility with an increase in the weight percentage of PANI [46]. The PVDF total crystallinity ($X_{c}$) was calculated by Equation (5) [47]:

(5) $$X_{c}=\frac{\Delta H_{f}}{\Delta H_{f}^{*}}\times 100\%$$

where $\Delta H_{f}$ is the melting enthalpy of PVDF measured by DSC and $\Delta H_{f}^{*}$ is the melting enthalpy for a 100 % crystalline PVDF (104.7 J g^−1^). By increasing the content of PANI from 10 to 50 *wt* % in the blends, the crystallinity of PVDF decreased from 55.1 % to 39.6 %, and the T_g_ of PVDF in the blend reduced from −42 to −50 °C. Despite the intermolecular hydrogen bonding between the components of blends through N-H (polyaniline) and C-F (PVDF) [48], the blends showed immiscibility. The local free volume increased in the polymer because of intramolecular interaction**s** of PVDF. This enhancement makes the mobility of chains more convenient [49] and reduced T_g_. The crystallinity of PANI30 is higher than PANI20 due to the balance between nucleation and crystal growth. While adding PANI to PVDF increases nucleation sites, it also inhibits the overall growth of crystals. However, PANI30 the interaction parameter with PVDF decreases from 0.3 to 0.1 compared to PANI20. This reduced interaction means that PVDF chains are less likely to interact with each other, allowing them to more easily form crystalline structures [50,51].

**Table 1:** T_g_, T_m_, ΔH_m_, $\upsilon_{1}$, *χ*, and X_c_ for different blend films.

Sample	T_g__PVDF_ (°C)	T_m PVDF_ (°C)	T_g__PANI_ (°C)	ΔH_m_ (J g^−1^)	$\upsilon_{1}$	*χ*	X_c_ (%)
PANI10	−50.8	171.6	52.2	57.7	0.08	15.1	55.1
PANI20	−50.3	174.6	55.8	42.8	0.16	5.4	40.9
PANI30	−50.1	172.5	50.7	48.4	0.25	1.6	46.2
PANI40	−49.7	172.2	47.4	40.2	0.34	0.8	38.3
PANI50	−50.8	173.0	42.3	41.7	0.44	0.5	39.6

The thermal stability of the blends before and after swelling was estimated from TGA as demonstrated in Fig. 2d–e and Table 2. Increasing content of PANI in the blend resulted the decrease of initial decomposition temperature (T_d,5 %_) from 370.0 to 339.1 °C for PANI10 to PANI50. This is due to the lower onset of degradation of PANI [52] compared to PVDF [53]. On the other hand, with the decrease of PVDF, the ash content decreased from 26 to 14 *wt* %. The T_d,5 %_ of blends decreased after swelling due to the presence of solvent; on the other hand, DTG gives two peaks. The first peak (T_d,max1_) is related to the solvent [54] and the second peak (T_d,max2_) was associated with the blend degradation. The T_d,max2_ increased after swelling in lithium salt solution from 376.6 to 484.4 °C for PAN40. Although the formation of complex between ionic salt and the backbone of chains reduces thermal stability, PANI40 and PANI50 showed higher thermal stability [55].

**Table 2:** Ash, T_d,5 %_, and T_d,max_ before and after swelling for PVDF/PANI blend films.

Sample	Before swelling			After swelling			
	Ash (%)	T_d,5 %_ (°C)	T_d,max_ (°C)	Ash (%)	T_d,5 %_ (°C)	T_d,max1_ (°C)	T_d,max2_ (°C)
PANI10	26.8	370.0	503.3	24.1	173.9	177.1	503.9
PANI20	23.9	364.1	500.8	21.0	170.8	176.8	496.5
PANI30	20.7	350.8	493.3	18.5	176.9	176.9	493.1
PANI40	17.4	345.0	376.6	15.0	168.6	178.1	484.4
PANI50	14.1	339.1	376.6	12.3	170.8	177.6	478.5

### 3.2 Electrochemical properties of blend films

**Fig. 3:**
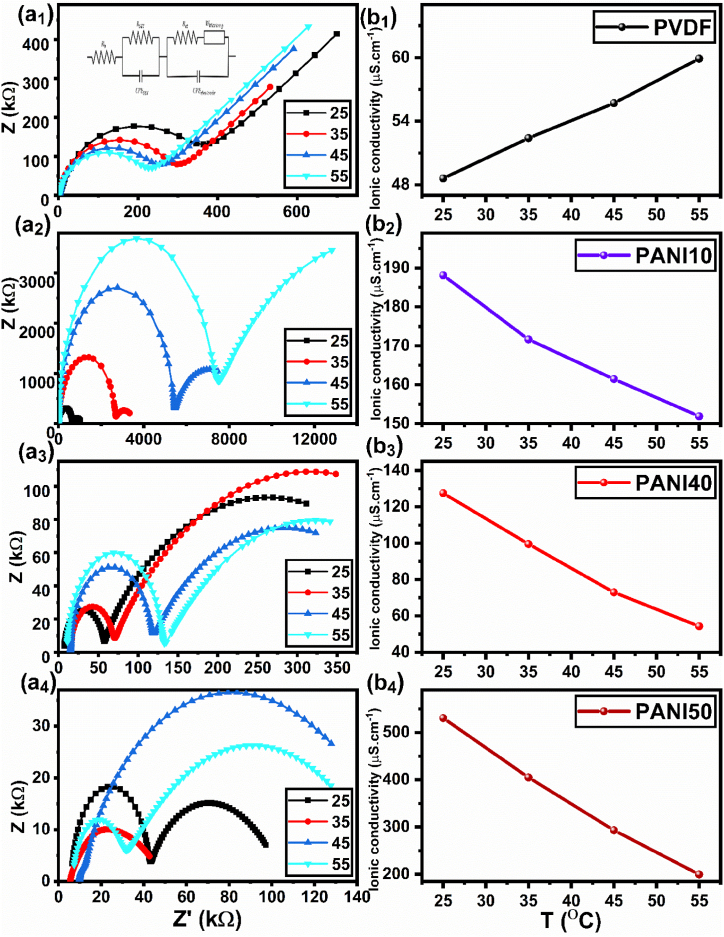
(a) EIS results of doped blends by lithium salt, (b) Ionic conductivity of samples at different temperature.

**Fig. 4:**
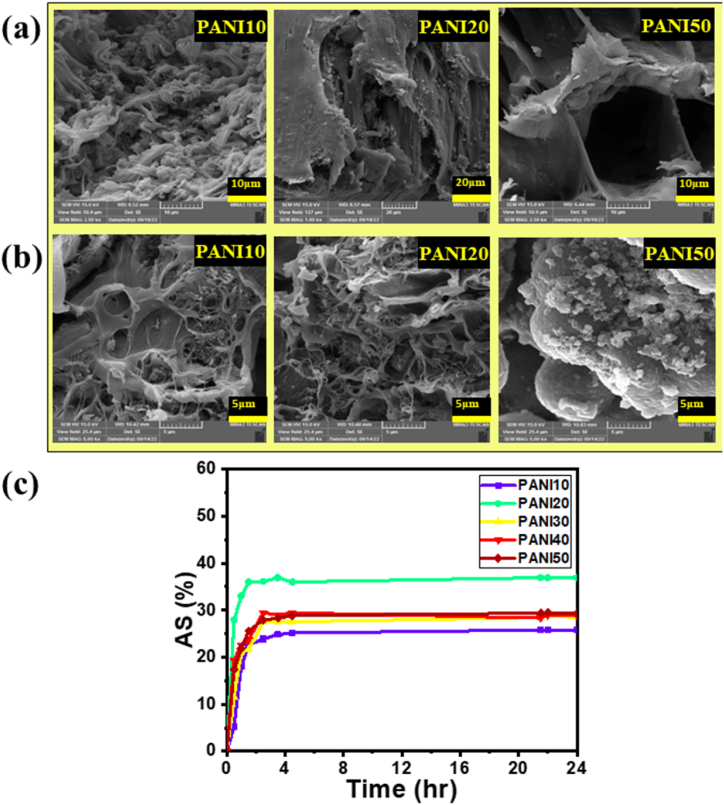
Morphology of polythiophene/PVDF blends (a) before and (b) after swelling in lithium salt solution, and (c) Electrolyte absorption by blends.

The ionic conductivity of the prepared samples was measured by EIS and results were fitted with an equivalent circuit (Fig. 3). According to the results of fitted RC circuit (R_SEI_, W), the ionic conductivity was calculated according to Equation (6) [56] at 25, 35, 45, and 55 °C and the results are shown in Table 3.

(6) $$\sigma =\frac{h}{R_{b}A}$$

where *σ* is the ionic conductivity (S cm^−1^), *h* is the thickness of sample (cm), *R*_*b*_ is the bulk resistance (Ω), and *A* is the area of the electrode/electrolyte interface (cm^2^). According to Table 3, the R_SEI_ of PANI10 has increased from 0.62 to 7.2 MΩ when the temperature increased from 25 to 55 °C. However, the R_SEI_ value of PVDF decrease from 0.28 to 0.18 (MΩ) under the same conditions. The R_SEI_ of the blends also increased with increasing temperature. This could be due to side reactions [57]. Although stainless steel as electrode has good chemical resistance and compatibility with electrolyte, the side reaction happened between carbonate solvent and lithium salt [58,59]. As the PVDF content decreased in the samples, the crystallinity of the blends decreased, which led to an increase in ionic conductivity from 4.9 × 10^−5^ to 5.3 × 10^−4^ S cm^−1^ for PVDF to PANI50. This indicated the improvement of ion mobility in the polymer matrix. On the other hand, a higher PVDF ratio in the electrolytes had the opposite effect; it made the blends more microviscous, which hindered the charge carriers and lowered the conductivity [60]. The ionic conductivity of PVDF increased from 4.9 × 10^−5^ to 6.0 × 10^−5^ S cm^−1^ S.cm^−1^ with increasing the temperature from 25 to 55 °C, which could be attributed to the higher chain mobility [61]. This enhancement might be assigned to two phenomena. Firstly, it increased the free volume of the system and secondly, it provided a pathway for ion transport [62]. The ionic conductivity depends on morphology of electrolyte. According to FE-SEM images (Fig. 4), at lower PANI concentrations in the blend, droplets are observed in the morphology. However, at higher concentrations, such as in PANI40, the morphology changes to a co-continuous structure. PANI50 demonstrates higher ionic conductivity, which is related to the higher ionic conductivity of PANI and the co-continuous structure at PANI50. On the other hand, co-continuous morphology makes passing tunnels for ion transport [63]. However, the ionic conductivity of the blends showed a decreasing trend with increasing temperature. This could be caused by the interaction between the fluorine group in PVDF and the N-H group in PANI [64]. This could be caused by decreasing the hydrogen bonding that resulted in formation of complex between lithium ion and polymers. Warburg (W) show how the ions move in the solid part inside the active material like cathode. The W element also shows how the speed of the electric force affects the ion movement. The W value of the pure PVDF compared to the blends has decreased from 21.0 to 2.0 × 10^−6^ (Ω), which indicated that the solid diffusion of lithium ion has improved This means the charge can move faster and easier in the blend (PANI10,20,30,40 and 50) than in the pure PVDF material [65].

**Table 3:** Electrochemical properties of PANI/PVDF blend electrolytes.

Samples	Temperature (°C)	R_SEI_ × 10^5^(Ω)	Ionic conductivity (mS.cm^−1^)	W (μΩ)
PVDF	25	2.8	0.049	21.0
	35	2.5	0.052	31.0
	45	2.0	0.056	28.0
	55	1.8	0.060	20.0
PANI10	25	6.2	0.188	0.1
	35	26.4	0.172	0.6
	45	54.0	0.161	0.2-
	55	73.0	0.152	0.1
PANI20	25	0.2	0.215	5.2
	35	0.2	0.162	4.7
	45	0.5	0.112	2.5
	55	1.6	0.072	1.1
PANI30	25	0.1	0.371	8.9
	35	1.2	0.260	8.0
	45	0.3	0.179	1.5
	55	0.4	0.083	6.7
PANI40	25	0.5	0.128	2.1
	35	0.5	0.100	2.1
	45	1.0	0.073	2.4
	55	1.2	0.054	2.8
PANI50	25	0.0	0.531	4.0
	35	0.4	0.405	1.8
	45	0.2	0.293	1.6
	55	0.8	0.200	1.4

According to Fig. 4a, FE-SEM images before swelling reveal immiscible morphology, where the continuous phase becomes partially miscible at higher PANI content in the blend [66]. In addition, a porous structure of the blends is observed that provides good transport of lithium ion [67,68]. Fig. 4b shows that grains are attached to the spherulite structure. These grains can attach to and aggregate the lithium salt at the interface of PANI, because of the complex formed between the secondary amine in PANI and the lithium salt [69]. As Fig. 4c demonstrates, the absorption swelling percentage (AS%) of all samples ranges from 25 % to 40 %. The reason for the low swelling is due to the solvent resistance of the crystalline domain of PVDF [70].

**Fig. 5:**
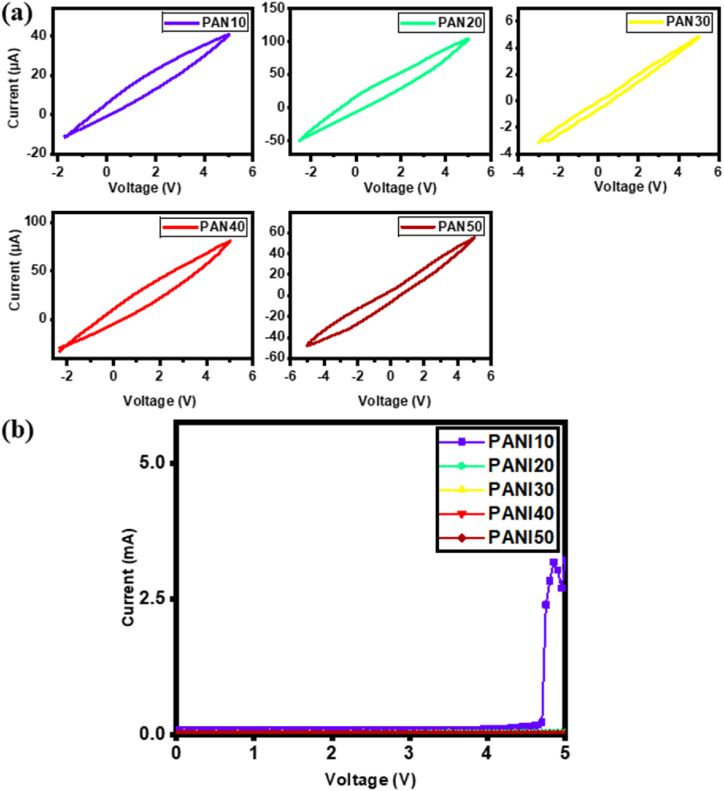
(a) Cyclic voltammetry test results, (b) LSV test of different samples.

As shown in the cyclic voltammetry (CV) curves in Fig. 5a the oxidation of LiCoO_2_ [71] is reflected by the oxidation peaks at 3.4, 3.8, 3.8, 4.1, and 4.3 V for PANI10, 20, 30, 40 and 50, respectively. The linear sweep voltammetry (LSV) results in Fig. 5b demonstrate that the electrochemical stability window (ESW) of PANI10 is about 4.6 V, while the ESW of PANI40 and 50 are higher than 5 V, due to the interaction between PANI and PVDF [72,73]. The high ESW of PANI40 and 50 makes them more suitable for lithium ion applications. In related studies, Mishra et al. [74] developed a GPE system by blending PVDF-HFP with PMMA and incorporating a non-aqueous liquid electrolyte containing NaCF_3_SO_3_. Their research focused on examining how the addition of PMMA to PVDF-HFP influenced the ionic conductivity of the GPE. Initially, the PVDF-HFP-based GPE exhibited an ionic conductivity of around 6.1 × 10^⁻⁴^ S cm^⁻1^. However, when PVDF-HFP was blended with PMMA in a 9:1 ratio, the ionic conductivity increased to approximately 7.5 × 10^⁻⁴^ S cm^⁻1^. This optimized blend showed enhanced ionic conduction and demonstrated sufficient electrochemical stability, with a stability window of about 3.6 V. Additionally, a porous gel polymer electrolyte was developed using a PMMA/PVDF blend, which was activated in a liquid electrolyte consisting of sodium tetrafluoroborate (NaBF_4_) dissolved in the ionic liquid 1-butyl-3-methylimidazolium tetrafluoroborate (BMIMBF_4_) [75]. The optimized membrane exhibited a porosity of 59 % and a liquid electrolyte uptake of 187 %, along with a maximum ionic conductivity of ∼0.8 mS cm^⁻1^. Additionally, the membrane demonstrated a Na^+^ ion transport number of 0.21 and an electrochemical stability window of around 5.5 V, highlighting its potential for advanced electrochemical applications [76].

**Fig. 6:**
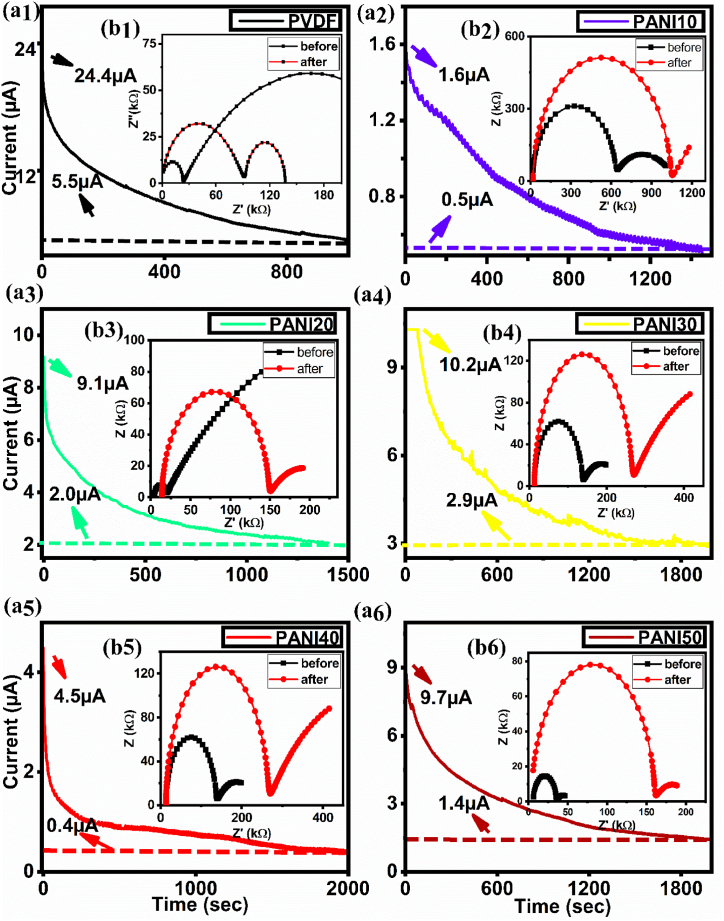
(a) Chronoamperometry and (b) EIS results to calculate cation transfer number.

The lithium-ion transference number (t _Li+_) was determined using the chronoamperometry test, as shown in Fig. 6. The t _Li_+ was calculated using Equation (7) [56]:

(7) $$t_{\;\text{Li}+}=\frac{I_{ss}\;\left(\Delta V-I_{0}\;R_{b.0}\right)}{I_{0}\left(\Delta V-I_{ss}\;R_{b.ss}\right)}$$

where *I*_*0*_ represents the initial current, *I*_*ss*_ represents the steady-state current, and *R*_*b.0*_ and *R*_*b. ss*_ represent the initial and final resistances, respectively, which were measured using AC impedance spectroscopy. Table 4 shows the results of t _Li +_ for different compositions of PANI/PVDF blend. The t _Li +_ decreased from 0.30 to 0.14 as the weight percentage of PANI increased from 10 to 50 %. The low t _Li_ + demonstrated strong interaction between cation and polymer matrix [77]. As the content of PANI increases, the number of N-H sites for lithium ion coordination also increases [78]. This results in the trapping or immobilization of the cations and more negative charge conducted [79].

**Table 4:** Bulk resistance of initial polarization, final polarization, and ion transfer number values of samples.

Sample	R_O_ × 10^5^(Ω)	R_s_ × 10^5^(Ω)	dv	t_+_%
PVDF	20.0	0.6	100000	30.0
PANI10	712.0	42450	100000	31.3
PANI20	88.4	156.2	100000	23.1
PANI30	11.9	16.4	100000	28.4
PANI40	10.2	4793.0	100000	18.9
PANI50	16.9	46.2	100000	14.4

**Fig. 7:**
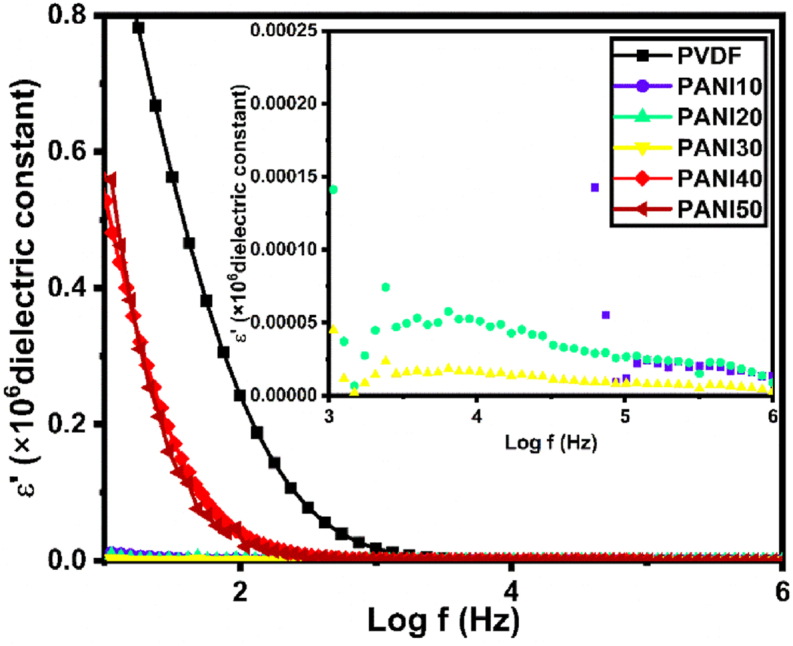
Dielectric constant versus frequency for different samples.

The dielectric constant was calculated using EIS results by Equation (8) [80]:

(8) $$\varepsilon_{r}=\frac{Z_{i}}{\omega C_{0}\left(Z_{r}^{2}+Z_{i}^{2}\right)}$$

where *Z*_*i*_, *Z*_*r*_, *C*_0_ and ω are the imaginary impedance (Ω), the real impedance (Ω), the vacuum capacitance in F ($C_{0}=\frac{\varepsilon_{0}A}{t}$ ($\varepsilon_{0}$ is the permittivity of free space equal $8.85\times 10^{-12}$, *A* is the surface area of sample in cm^2^, and *t* is the thickness of sample in cm)) and ω=2πf (*f* is the frequency in Hz). Fig. 7 shows the dielectric constants of PANI10, 20, 30, 40, and 50 samples as a function of frequency. The dielectric constant decreases rapidly at lower frequencies, while pure PANI has a nearly constant value [81]. This is attributed to the Maxwell–Wagner effect, which causes charge accumulation at the interface of the two materials due to the different relaxation times of the charge carriers [82,83]. As the PANI content increases from PANI10 to PANI50, the polarization effect also increases due to the enhanced interfacial interaction between the fluorine group in PVDF and the N-H group in PANI [84,85]. Due to its high dielectric constant, the pure PVDF sample has the highest transference number of Li^+^, which means it can dissolve more lithium salts. The high dielectric constant and the presence of the (-C-F) functional groups also contribute to the high electrochemical stability of PVDF based systems [86].

## 4 Conclusions

We synthesized polymer electrolytes based on blends of PANI and PVDF with different compositions. We characterized the morphology, crystallinity, ionic conductivity, transference number, and electrochemical performance of the samples. Our results revealed that the PANI/PVDF blends were immiscible, as observed in the FE-SEM images and interaction parameters. The addition of PANI acted as a plasticizer and reduced the crystallinity of the blends measured by DSC. At higher PANI content, the T_g_ decreased and T_m_ increased because of weakened intermolecular forces and the chain mobility, while the dispersed phase raised the T_m_ by hindering the chain movement and the crystallization. The thermal properties improved for the PANI40 and 50 blends. The ionic conductivity of the blends increased with the PANI content, reaching a maximum value of 5.3 × 10^−4^ S/cm for the blend with 50 *wt* % PANI. This was attributed to the decreasing crystallinity of the blend that enhanced chain mobility. The ionic conductivity of the PANI/PVDF blend decreased with increasing temperature, because of the increase in chain mobility and decrease in hydrogen bonding. So, the number of available N-H sites for formation of complex with lithium salt increased. Also, the cation transfer number of the PANI/PVDF blend decreased with increasing PANI content, due to the strong interaction and trapping of lithium ions by the N-H sites on PANI. In addition, the PANI20, PANI30, PANI40, and PANI50 blends demonstrated wide electrochemical stability windows that satisfied lithium battery applications.

## Funding

This work is not funded.

## Data availability

Data will be available on the request from corresponding author (Mehdi Salami-Kalajahi, m.salami@sut.ac.ir).

## CRediT authorship contribution statement

**Mahdi Kargar-Esfandabadi:** Writing – original draft, Investigation. **Marzieh Golshan:** Writing – original draft, Validation. **Hossein Roghani-Mamaqani:** Validation. **Mehdi Salami-Kalajahi:** Writing – review & editing, Supervision, Funding acquisition, Conceptualization.

## Declaration of competing interest

The authors declare that they have no known competing financial interests or personal relationships that could have appeared to influence the work reported in this paper.
